# An improved 2b-RAD approach (I2b-RAD) offering genotyping tested by a rice (*Oryza sativa* L.) F2 population

**DOI:** 10.1186/1471-2164-15-956

**Published:** 2014-11-05

**Authors:** Yu Guo, Hui Yuan, Dongming Fang, Lianbo Song, Yan Liu, Yong Liu, Lu Wu, Jianping Yu, Zichao Li, Xun Xu, Hongliang Zhang

**Affiliations:** Beijing Genome Institute-Shenzhen, Beishan Industrial Zone, Yantian District, Shenzhen, 518083 China; Beijing Key Laboratory of Crop Genetic Improvement, College of Agronomy and Biotechnology, China Agricultural University, Beijing, 100193 China; National Institute of Biological Sciences, Beijing, Zhongguancun Life Science Park, Changping District, Beijing, 100026 China

**Keywords:** 2b-RAD, Genotyping, Genetic linkage map

## Abstract

**Background:**

2b-RAD (type IIB endonucleases restriction-site associated DNA) approach was invented by Wang in 2012 and proven as a simple and flexible method for genome-wide genotyping. However, there is still plenty of room for improvement for the existent 2b-RAD approach. Firstly, it doesn’t include the samples pooling in library preparation as other reduced representation libraries. Secondly, the information of 2b-RAD tags, such as tags numbers and distributions, in most of species are unknown. The purposes of the research are to improve a new 2b-RAD approach which possesses samples pooling, moreover to figure out the characteristic and application potentiality of 2b-RAD tags by bioinformatics analysis.

**Results:**

Twelve adapter1 and an adapter2 were designed. A library approach comprising digestion, ligation, pooling, PCR and size selection were established. For saving costs, we used non-phosphorylated adapters and indexed PCR primers. A F2 population of rice (*Oryza sativa* .L) was genotyped to validate the new approach. On average, 2000332 high quality reads of each sample were obtained with high evenness. Totally 3598 markers containing 3804 SNPs were discovered and the missing rate was 18.9%. A genetic linkage map of 1385 markers was constructed and 92% of the markers’ orders in the genetic map were in accordance with the orders in chromosomes. Meanwhile, the bioinformatics simulation in 20 species showed that the *Bsa*XI had the most widespread recognition sites, indicating that 2b-RAD tags had a powerful application potentiality for high density genetic map. Using modified adapters with a fix base in 3′end, 2b-RAD was also fit for QTL studies with low costs.

**Conclusions:**

An improved 2b-RAD genotyping approach was established in this research and named as I2b-RAD. The method was a simple, fast, cost-effective and multiplex sequencing library approach. It could be adjusted by selecting different enzymes and adapters to fit for alternative uses including chromosomes assembly, QTL fine mapping and even natural population analysis.

**Electronic supplementary material:**

The online version of this article (doi:10.1186/1471-2164-15-956) contains supplementary material, which is available to authorized users.

## Background

Genotyping with the molecular markers - detecting the heritable polymorphisms among the individuals of one or more populations - are employed in many regions in modern biological research including phylogeny, evolution, plant breeding and disease research, etc. [1–3]. The classical molecular markers, such as restriction fragment length polymorphisms (RFLPs), randomly amplified polymorphic DNA (RAPD), amplified fragment length polymorphisms (AFLPs) and simple sequence repeats (SSRs) were proven to be powerful in genotyping. However, based on gel electrophoresis, these methods usually take long time and high labor costs with large samples size. In addition, the DNA polymorphisms with artificial bands counting are prone to error. Moreover, these methods generally produce limited markers, making it difficult to construct high density genetic map that is essential for chromosomes assembly. Therefore, the fast, cost-effective and high-throughput genotyping techniques are required.

Now, with the advent of next-generation sequencing (NGS), there are several such approaches, which are capable of genotyping not hundreds but thousands of markers in a single step [4]. Some newly developed techniques, combining NGS with restriction enzymes (REs) fragments, are widely used for genotyping. These techniques internally sequence the regions around REs recognition sites; produce a reduced representation of a genome. The restriction-site associated DNA (RAD) technique is capable of sequencing the regions adjacent to recognition site of a chosen restriction enzyme and widely applied to detecting single nucleotide polymorphisms (SNPs), even insertions/deletions (InDels), for quantitative trait locus (QTL) or evolutional analysis [5–8]. With a simple, quick library approach and lower coverage requirement, genotyping-by-sequencing (GBS) is particularly well suited for genotyping populations with large samples sizes [9–11]. Two-enzyme GBS and double digest RAD sequencing are the other two reduced representation libraries both based on simultaneous double REs digestion, which are also used for SNP discovery and genotyping [12, 13].

The 2b-RAD (type IIB endonucleases restriction-site associated DNA) approach relies on the type IIB REs, such as *Bsa*XI or *Alf*I (both insensitive enzyme for methylation), to produce uniform tags. It was proven to be a simple and flexible method for genome-wide genotyping [14]. Poland JA et.al compared many reduced representation libraries and concluded that 2b-RAD approach had the advantages of producing uniform length tags, allowing nearly all of the restriction sites to be surveyed and permitting marker intensity adjustment [15]. However, there is still plenty of room for improvement of the existent 2b-RAD approach. Firstly, it is not as multiplex as other reduced representation libraries using barcode adapter and pooling procedure. By using barcode adapters, many samples equal to one sample to carry on the subsequent library procedures after pooling. Therefore, it can save much time and labor cost. The RAD’s multiplexing level achieves 96 samples while the GBS achieves 48 up to 384, for instance [15]. The existent 2b-RAD approach achieves its multiplexing sequencing by using indexed PCR primers. The disadvantage is that each sample is prepared to a single sequencing library. It takes more times and labor cost, and is not fit for the populations with large sample size. Lacking of fast library approach would limit its application. Secondly, the information of 2b-RAD tags, such as tags numbers and distributions, in most of species are unknown except *Arabidopsis thaliana* [14]. In order to make better use of it, it is necessary to do a comprehensive testing.

Based on the above reasons, firstly, we tried to improve the existent 2b-RAD library approach. At the present times, multiplex sequencing methods have been developed for NGS using either barcoded adapters, especially in reduced representation library [5–13], or indexed amplification primers [16–19]. We attempted to combine these two methods together to develop a new multiplexing 2b-RAD library approach. Twelve adapters with different barcodes were designed in this research and 12 samples could be pooled together. To validate the new approach, we prepared 24 2b-RAD libraries containing 285 samples from a rice (*Oryza sativa* .L) F2 population. The population was genotyped and a genetic linkage map was constructed. The markers were aligned with Nipponbare reference to measure the accuracy of the genetics map. Moreover, series of bioinformatics simulate analysis of 2b-RAD tags based on ten plants and ten animals’ reference genomes were carried out. Processing with methylation-sensitive REs, it is difficult to predict which recognition sites would be digested in GBS. So we only compared RAD and 2b-RAD tags on these species. The tag numbers and distributions were analyzed specifically.

## Methods

### Comparison of RAD and 2b-RAD tags

The REs recognition sites, including *Eco*RI, *Sbf*I and *Hin*dIII for RAD, *Bsa*XI and *Alf*I for 2b-RAD, were detected on the reference genomes containing 10 plants and 10 animals. Each sequence of “GAATTC” (*Eco*RI), “CCTGCAGG” (*Sbf*I) and “AAGCTT” (*Hin*dIII) tested on the genomes was regarded as one recognition site, producing two tags for RAD [5]; each sequence of “ACNNNNNCTCC”, “GGAGNNNNNGT” (both for *Bsa*XI) and “GCANNNNNNTGC” (*Alf*I) was regarded as one recognition site, producing one tag for 2b-RAD, respectively.

The *Eco*RI was one of the most commonly used REs for RAD. For 2b-RAD, the recognition sites of *Bsa*XI were much more than *Alf*I by simulation. The recognition bases of *Eco*RI and *Bsa*XI were both six. So the application potential of *Eco*RI and *Bsa*XI were compared. The genomes size was divided by *Eco*RI sites numbers. The average fragment length was approximately 4000 bp of the 20 species, indicating that *Eco*RI recognition sites located once per 4000 bp. Each 4000 bp sequence was regarded as a window and represented a simulated tiny scaffold. The recognition sites equaled 0, 1 and more than 1 in these scaffold denoted different potentiality of assembly. Zero indicated that the scaffold was impossible to be connected to any chromosome; one indicated the scaffold could be connected with no orientation; more than one indicated potential perfect assembly with an orientation. The sites of *Eco*RI and *Bsa*XI were detected in these tiny scaffolds to estimate their potentiality for chromosome assembly.

### F2 populations

The F2 population materials including two F0 parents and 277 F2 progeny were gained from the rice genetic breeding laboratory of China Agricultural University. Of the two parents, the female was Nipponbare while the male was a stable recombinant inbred line for five generations of *Oryza sativa* spp. *Japonica* line whose ancestry derived from a cross between two *Japonica* varieties. One was Nipponbare, and the other was a Chinese landrace with the name of Mayi danru. The parents and their progeny, altogether 279 DNA were extracted from their fresh leaves according to Doyle’s protocol [20]. The DNA only with a lowest concentration of 50 ng/μL and no degradation were able to be applied for the following 2b-RAD library.

### Adapter design

To create a simple and quick 2b-RAD library approach with pooling procedure, we designed two kinds of adapters. Adapter1 with 5–9 bp barcodes was complementary to the Illumina multiplexing PCR primer 1.0; adapter2 was complementary to index primer. The digested fragment of *Bsa*XI was 33 bp with 3 bp random overhangs on the 3′ ends. The sequences of the adapter1 were:

5′-ACACTCTTTCCCTACACGACGCTCTTCCGATCTxxxxxNNN-3′ and 5′-yyyyyAGATCGGAAGAGCGTCGTGTAGGGAAAGAGTGT-3′, the “xxxxx” and “yyyyy” denoted the barcode and its complement sequences. The NNN was complementary to the 3 bp sticky end generated by *Bsa*XI (“N” was a random base of A, G, C, T and there were 64 kinds of combinations altogether). The adapter2 was:

5′- AGATCGGAAGAGCACACGTCTGAACTCCAGTCAC -3′ and 5′-GTGACTGGAGTTCAGACGTGTGCTCTTCCGATCTNNN-3′. The annealed adapter1 and adapter2 were adjusted to 5 μM as working concentration.

The *Bsa*XI digested fragment was only 33 bp and it would be completely sequenced by single-end 50 bp (SE 50), so a little longer (9 bp) barcodes would not reduce the tag sequence. The barcodes were designed according to Poland JA’s criteria [12] and made slight modification. (1) The lengths of barcodes were different form 5 to 9 bp to maximize the balance of bases at each position, especially in the *Bsa*XI recognition sites. (2) The barcodes must be two or more bp different from all other barcodes. (3) The barcodes can’t contain or recreate (after ligation step) *Bsa*XI restriction site. A set of 12 barcode sequence were designed (Additional file 1).

### 2b-RAD library preparation

The concentrations of all DNA samples were adjusted to 50 ng/μL. The DNA (200 ng) was digested in 10 μL reaction volume of NEB Buffer 4 with 1 U *Bsa*XI (New England BioLabs Inc, Catalog # R0609L) at 37°C for 2 h. An additional DNA was digested simultaneously to detect the digestion efficiency by 1% agarose gel electrophoresis. The primary DNA band disappeared and became disperse, indicating a successful digestion. Then the ligation reaction was completed in the same tube as the digestion, combining the remaining digested DNA with 2 μL of the adapter1, 2 μL of the adapter2, 2 μL ligase buffer and 400 U of the T4 ligase (New England BioLabs Inc, Catalog # M0202L). *Bsa*xI could not be inactivated by heating, so the digested productions was recommended performing the ligations in 4°C for 1 hour, then hold on ice [14].

For exploring the relationship between sequencing data size and enzyme sites coverage, each parents repeated 4 times in library experiment. All 285 DNA samples (277 progeny +4 female +4 male) were divided into 24 groups. Each group contained 12 samples and the last group contained 9 samples. The samples in each group were ligated with different 12 adapters.

Twelve samples in a group were pooled together when they were purified using purification kit (QIAquick PCR Purification Kit, Catalog # 28106). The ligated productions were completely combined to keep the amount of pooled DNA as 1 ~ 1.5 μg, which was sufficient for the following PCR procedure. The samples in each group with different barcode adapters were gathered to a tube which had been added the wash buffer of purification kit beforehand. Then the pooled DNA was purified according to the manufacturer’s instructions and eluted in 25 μL EB (elution buffer).

The Illumina multiplexing PCR primers were used for amplification. The sequences were:

5′-AATGATACGGCGACCACCGAGATCTACACTCTTTCCCTACACGACGCTCTTCCGATCT-3′ (multiplexing PCR primer 1.0) and

5′-CAAGCAGAAGACGGCATACGAGATXXXXXXGTGACTGGAGTTCAGACGTGTGCTCTTCCGATCT-3′ (index primer), where the six “X” represented a 6 bp index. The index primer sequences were derived from a BGI patent (http://www.google.ca/patents/WO2012037880A1?hl=zh-CN&cl=en) [21]. For each PCR, we combined 50 ng of pooled DNA, 1 μL of each primer (10 μM), buffers, nuclease-free water and Phusion polymerase (New England BioLabs Inc, M0531) at a final 50 μL total volume. Temperature cycling consisted of 98°C for 30 seconds followed by 12 cycles of 98°C for 30 seconds, 65°C for 30 seconds, 72°C for 30 seconds with a final *Taq* extension step at 72°C for 5 minutes. Four kinds of index primers were used in this study for distinguishing the libraries (Additional file 1). The expected tag fragment after PCR was from 160 bp to 164 bp (5–9 bp barcode). PCR production was detecting by 2% agarose gel electrophoresis, and then the 150–200 bp bands were cut and purified by the purification gel kit (QIAquick Gel Extraction Kit, 28704) and eluted in 30 μL EB.

The quantify molarity and library fragment size distribution of cleaned DNA were detected by an Agilent Bioanalyzer. Quantification was conducted by qPCR. SE50 sequencing of four 12-plex libraries per flowcell channel lane was performed on Hiseq 2000 platform (Illumina, Inc.). Totally six lanes were used.

**Figure 1:**
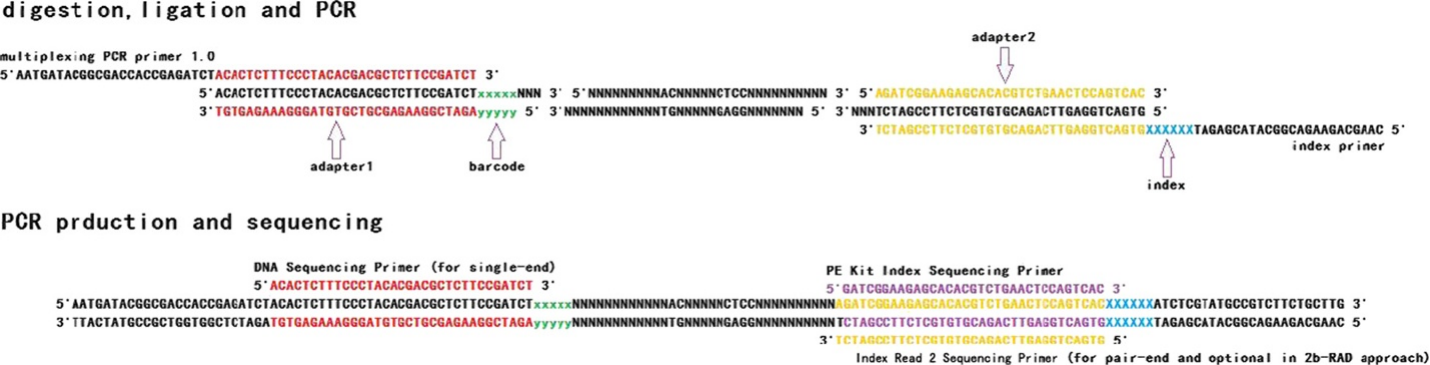
**The complementary relationships of all the sequence.** The same color represented complementation.

The detailed protocol of whole 2b-RAD library procedure was available on Additional file 2. The complementary relationships of all the sequence used in 2b-RAD library were showed on Figure 1.

### Filtering raw sequence data

The reads filtering steps were performed by our own Perl scrip as follows. (1) Matched one of the 12 barcodes allowing one mismatch. After the reads were assigned into each sample, the 5–9 bases barcode were removed. (2) The reads following on the heels of the barcode should perfectly matched *Bsa*XI fragment “NNNNNNNNNNNNACNNNNNCTCCNNNNNNNNNN” or “NNNNNNNNNNGGAGNNNNNGTNNNNNNNNNNNN” (these “N” means random sequences of *Bsa*XI digestion fragments) with no “Ns” (“Ns” means the bases which were failed to be sequenced by Hiseq 2000). Then the reminder bases of each read were deleted. Two kinds of 33 bp tags containing the recognition sites “ACNNNNNCTCC” or “GGAGNNNNNGT” were obtained. They were regarded as high quality reads.

**Figure 2:**
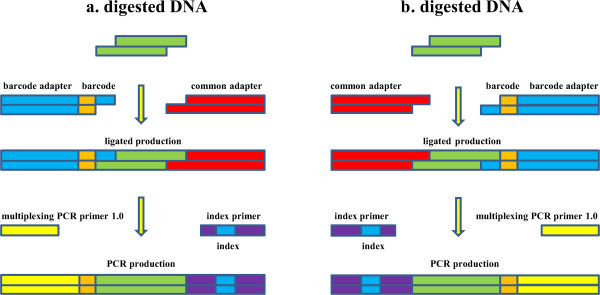
**The tag with many copies which was probably sequenced both form plus strand and minus strand.** The **a** and **b** revealed that the adapter1 and adapter2 may ligate to any ends of the 33 bp digested fragment and the tag would be sequenced from any 5′ end.

The two cohesive ends of *Bsa*XI digestion fragment were identical, so the adapter1 was probably ligated to any ends of the fragment, as well as the adapter2. So a fragment with many copies after DNA extracting and digestion, was ligated to adapters of couple of possibilities. However, only the fragment which was ligated adapter1 and adapter2 simultaneously could be PCR amplified. The fragment with only 33 bp length, was probably sequenced both form plus strand and minus strand (Figure 2). In order to solve this problem, all the tags contained “GGAGNNNNNGT” were translated to their reverse compliment sequences, namely, the form of “ACNNNNNCTCC”. And then the tags contained only one strand of the digestion fragments.

### The tag mapping

For estimating *Bsa*XI sites coverage, firstly, simulant detections of the *Bsa*XI sites in Nipponbare reference genome were carried out. The 33 bp fragments in reference containing recognition sites of “GGAGNNNNNGT” or “ACNNNNNCTCC” were picked out. Then these sequences were mapped to reference using Bowtie (version 0.12.7) with the parameter of -m 1 -v 2 allowing 2 mismatches. The sequences which aligned with only one location were regarded as unique tags and the expected potential markers.

Secondly, the high quality reads of 285 samples were mapped to Nipponbare reference (Bowtie -m 1 -v 1). The mapped reads were detected whether they covered simulant tags.

To look for the relationship between sequencing data size and *Bsa*XI sites coverage, a testing was performed based on several combinations of 4 repeated female samples. The female was Nipponbare, as well as the reference genome. Therefore the testing could accurately reflect the relationship.

After digestion, the fragments between two recognition sites also possessed sticky end “NNN” as follows. 

The structure made the fragments possible to be ligated with adapters and performed PCR amplification. If their length was nearly 33 bp, they would not be removed in the agarose gel size selection and be sequenced. So the reads matched barcodes while not matched *Bsa*XI recognition sites were analyzed. After removing redundant sequences (barcode and part of atapter2), they were mapped to Nipponbare reference genome (Bowtie -m 1 -v 1).

### SNP calling with and without reference

Any two of the female samples combination could cover nearly 100% of the simulant REs sites, so we combined female-2 and female-3 together (with the lowest data size) to perform SNP-calling. Same treatment to male samples of male-2 and male-4 were performed.

The SNP calling without references were performed using Stacks (version 1.01) [22] with four programs. The parameters were -m 2 -M 2 -N 1 for ustacks, -n 2 for cstacks, default for sstacks and -m 2 -t F2 -o joinmap -c --min_hom_seqs 2 --min_het_seqs 0.010 --max_het_seqs 0.011 for genotypes. The “-m” in genotypes means minimum reads depth for genotype. We used –m 2 as the depth and the automated corrections system (-c) in consideration of the lower sequencing depth of the progeny. The markers of aa x bb style were selected for genetic map. Meanwhile, the female tags of these markers were mapped to Nipponbare (Bowtie -m 1 -v 1).

To construct the genetic linkage map, the markers derived from Stacks were pretreated. The markers with less than 20% missing rate (<54 samples) were preserved. The linkage analysis was performed using JoinMap (version 4.1, http://www.Joinmap.nl). The SNP genotypes in F2 population were expected to segregate at a 1:2:1 ratio. Distorted markers (P <0.01) were filtered by χ^2^ test. The parameters using JoinMap were “independence LOD” for grouping method, “regression mapping” for mapping algorithm, “Kosambi’s” for mapping function method. The groups with less than 5 markers were discarded. Each linkage map marker’s location in alignment result was retrieved to measure accuracy of the genetic map. Of the sorted markers via genetic distances in each group, the one which was significantly opposite the mainstream of the order (more than 2Mbp to its adjacent two markers) was regarded as “order error” marker.

To roughly evaluate SNP numbers between two parents, the other way for genotyping relied on reference genome. Firstly, reads of two parents were mapped to the Nipponbare reference using SOAP2 (version 2.21) with default. The aligned reads were performed SNP calling using SOAPsnp (version 1.03) with default. The SNPs in cns file (SOAPsnp result file) were preserved only when the quality score of consensus genotype were more than 10. After integrating their SNP results, the genotype of aa x bb style between two parents were obtained. The two results between SOAPsnp and Stacks were compared.

## Result

### Comparison of RAD and 2b-RAD tags

The REs recognition sites of RAD and 2b-RAD in simulated species were listed in Table 1. The tag of RAD was twice the number of its recognition sites. On average, the *Bsa*XI had the most recognition sites numbers among the five REs. However, the tag numbers of *Eco*RI and *Hin*dIII were more than *Bsa*XI. The recognition sites of *Alf*I were much less than *Bsa*XI, making it no significance applications in high density linkage map, so we only used *Bsa*XI in the following simulation and experiment.

**Table 1:** **The REs recognition sites and tag numbers of RAD and 2b-RAD in simulated species**

Species	Size (G)	***Eco***RI	***Sbf***I	***Hin***dIII	***Bsa***XI	***Alf***I	Version
*Arabidopsis thaliana*	0.12	37057	627	67057	39826	12601	TAIR9
*Cucumis sativus*	0.24	69944	1197	94344	65249	23762	v2.3
*Theobroma cacao*	0.33	90253	1572	173770	81810	39800	CIRAD_v0.9
*Oryza sativa*	0.37	88471	5541	110084	180848	83465	IRGSP-7.0
*Vitis vinifera*	0.49	143785	2594	228694	155893	62070	Genoscope
*Cajanus cajan*	0.61	161249	1652	232261	162320	62119	v1.0
*Solanum tuberosum*	0.73	233925	2957	273063	212095	70042	v3.4
*Sorghum bicolor*	0.74	200543	11693	308083	379985	151244	phytozome_v7.0
*Glycine max*	0.97	309451	3844	422507	289940	140204	phytozome_v7.0
*Zea mays*	2.06	490315	50773	768456	1151014	444673	AGI_5a
Total	6.65	1824993	82450	2678319	2718980	1089980	
Average	0.67	182499	8245	267831	271898	108998	
Average tag number		364998	16490	535663	271898	108998	
*Drosophila melanogaster*	0.17	44863	2948	45913	58189	50403	RGSC3.4
*Apis mellifera*	0.23	97174	551	49977	58053	21237	AnoCar2.0
*Anopheles gambiae*	0.27	53807	3164	87297	100574	80148	Galgal4
*Takifugu rubripes*	0.39	75238	20718	95182	210480	103943	FUGU4
*Bombyx mori*	0.48	112113	2041	121288	96793	47329	AgamP3
*Gallus gallus*	1.05	277001	48010	403125	453890	400359	NCBIM37
*Danio rerio*	1.41	255801	30677	427420	426194	339437	v1.0
*Mus musculus*	2.72	758802	60302	842075	1331056	607106	2.0
*Rattus norvegicus*	2.72	817664	58283	818955	1257212	595706	r5.27
*Homo sapiens*	3.10	778227	77095	837133	1292381	583536	v2.0
Total	12.54	3270690	303789	3728365	5284822	2829204	
Average	1.17	327069	30378	372836	528482	282920	
Average tag number		654138	60758	745673	528482	282920	

The average value that genome size divided by *Eco*RI recognition sites number was 3647 bp in plants and 3833 bp in animals. We employed 4000 bp as a window to detect the presence numbers of *Eco*RI and *Bsa*XI recognition sites. Overall, 4749893 windows were detected. The windows where *Eco*RI recognition sites numbers equaled 0, 1 and more than 1 were 1839924 (39%), 1566583 (33%) and 1343386 (28%). The equivalents for *Bsa*XI were 1198293 (25%), 1340862 (28%) and 2210738 (47%).

In addition, we detected the recognition sites numbers of *Bsa*XI in the windows where *Eco*RI recognition sites numbers were 0 or 1. Zero or one meant that these windows were impossible to be perfectly assembled to a chromosome. There were 825203 (*Eco*RI =0 and *Bsa*XI >1) and 1218063 windows (*Eco*RI =1 and *Bsa*XI >0), and the percentages were 45% (825203/1839924) and 78% (1218063/1566583). The simulations of *Eco*RI =0, *Bsa*XI >1 and *Eco*RI =1, *Bsa*XI >0 both indicated that the windows contained more than one REs recognition sites. This fact revealed that the perfect assembled windows were significantly increased after adding the *Bsa*XI tags. The windows contained at least two recognition sites was up to 71.3% of the total windows.

**Figure 3:**
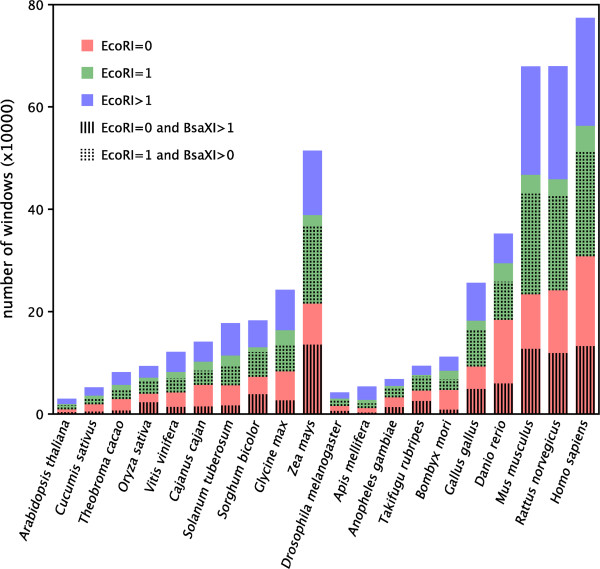
**The assemble potentiality simulation of** ***Eco*** **RI and** ***Bsa*** **XI in 20 species.** The different color represents the different windows where *Eco*RI recognition sites were 0, 1 or more than 1. The small figure of vertical lines represented the windows where *Eco*RI recognition sites were 0 and *Bsa*XI were more than 1. The small figure of spots represented the windows where *Eco*RI recognition sites were 1 and *Bsa*XI were more than 0.

The detailed windows simulation was showed on Figure 3 and Additional file 3.

### Experimental 2b-RAD results

Overall, the 24 libraries of 6 lanes produced 860368972 raw reads, equaling to 43 Gbp raw data. Each lane produced 7.2 Gbp data on average. The number of the reads matched barcode was 813497032 (94.6%). The reads matched both barcode and *Bsa*XI recognition sites were 570094697 (66.3%). These reads were regarded as high quality reads, including 305405977 (53.6%) reads which were the style of “ACNNNNNCTCC” and 264688720 (46.4%) reads which were “GGAGNNNNNGT”. The 264688720 reads were translated to the style of “ACNNNNNCTCC” before genotyping. Totally 290274275 (33.7%) raw reads were filtered. Of these filtered reads, 46817940 (16.1%) were filtered on account of barcode match failure and 243402335 (83.9%) were filtered by *Bsa*XI sites match failure.

**Figure 4:**
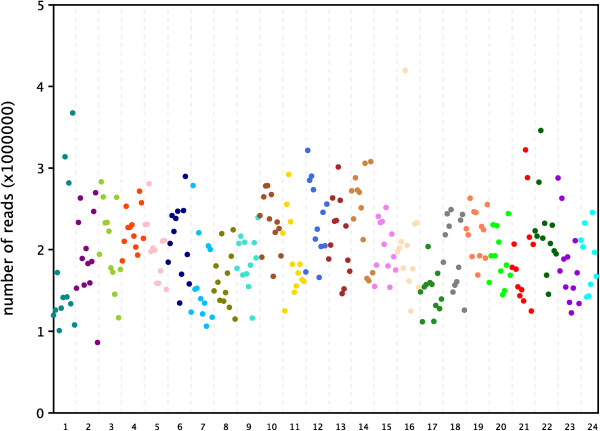
**The high quality reads numbers of the 285 samples.** The different colors represent different libraries.

Each sample’s reads divided by barcode were from 1815973 to 5303555. The ratio of the maximum divided by minimum was 2.9. After *Bsa*XI sites matching step, the high quality reads were from 862169 to 4195194, and the ratio was 4.9 (Figure 4). On average, each sample was given 2000332 high quality reads, equaling to 66 Mbp.

The detailed reads data of 285 samples and 24 libraries were listed in Additional file 4.

### Comparison of observed and expected 2b-RAD tag

The total expected *Bsa*XI sites in Nipponbare reference were 180848, including 105142 unique sites. All together 570,094,697 high quality reads of 285 samples were mapped to Nipponbare reference. The 550108260 (96.5%) reads could be aligned with the Nipponbare reference. On average, these reads covered 82.5% (149270) of the total expected *Bsa*XI recognition sites and 84.2% (88503) of the unique sites (Additional file 5). The actual average depth of each *Bsa*XI site was 10.67 (550108260/285/180848).

The combinations of 4 repeated female samples’ results were showed in Table 2.

**Table 2:** **The combinations of 4 repeated female**

Reads number	Depth	Combination	Covered sites	Coverage rate
2333752	12.8	fe-1	151387	83.71%
2225621	12.2	fe-2	151352	83.69%
1575530	8.5	fe-3	150720	83.34%
2456057	13.4	fe-4	156650	86.62%
3909282	21.6	fe-3 + fe-1	178654	98.79%
3801151	21.0	fe-3 + fe-2	177239	98.00%
4031587	22.3	fe-3 + fe-4	178946	98.95%
6134903	33.9	fe-3 + fe-1 + fe-2	180098	99.59%
8590960	47.5	fe-3 + fe-1 + fe-2 + fe-4	180104	99.59%

“fe” means female parent; “reads number” means the reads which were able to be mapped to reference; “depth” equals to reads number divided by the total expected *Bsa*XI recognition sites ; “covered sites” means the *Bsa*XI sites in reference which was covered by high quality reads; “coverage rate” equals to the “covered sites” divided by the total expected *Bsa*XI recognition sites.

The 243402335 filtered reads by *Bsa*XI sites matching step were also mapped to reference. The result revealed that 209287752 (86.0%) of these reads could be aligned with reference. Namely, 72.1% of the total filtered reads were able to be mapped to reference but failed to match *Bsa*XI recognition sites. This fact may indicate that these reads were the fragments between two *Bsa*XI recognition sites.

### Genotyping and genetic mapping

**Figure 5:**
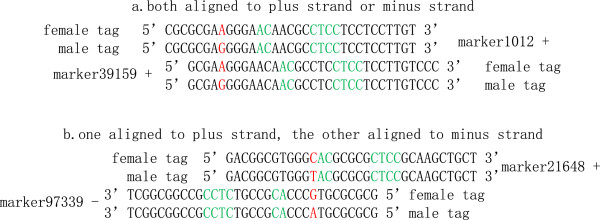
**The markers had overlaps.** The **a**, **b** revealed different conditions of strands between overlapped markers. The “marker1012, marker39159, marker21648, marker97339” were the marker ID; the “+” or “-” was the plus strands alignment or minus strands alignment; the “female tag” or “male tag” was the sequence of the marker in female parent or male parent. The red bases revealed SNPs; the green bases revealed *Bsa*XI recognition sites.

In the cstacks procedure, 118289 catalogs were produced by the two parents’ reads. After genotypes procedure, the markers of aa xx bb style were 3598 and all of them could be successfully aligned with reference including 3580 unique markers. Eighteen markers were mapped to more than one location. For the 3580 unique markers, there were 239 markers each contained two SNPs. The alignment result revealed another fact that 33 pairs of markers had overlaps. These overlaps were attributed to two close *Bsa*XI recognition sites in a chromosome (Figure 5). The probability of the overlapped makers was 0.9% (33/3598). Totally 3804 SNPs were confirmed. The SNPs yielded by SOAPsnp were 4769. There were 2633 SNPs loci were both in SOAPsnp result and Stacks results. All of the markers information was showed in Additional file 6.

**Figure 6:**
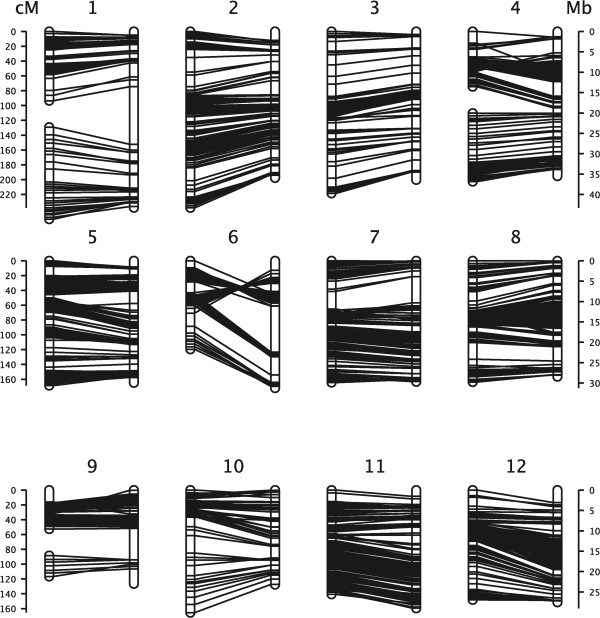
**The 1384 markers of 15 groups which were able to be aligned with Nipponbare reference.** The left vertical line was the linkage group and the right was the reference chromosome.

For the 3598 markers, the total missing rate was 18.9% (total missing alleles divided by 3598 × 277). The markers with less than 20% missing rate (<54 samples) were 2547. By χ^2^ test, 1391 markers survived. Six markers were assigned to some tiny groups (markers were less than 5). The reminder 1385 markers were divided into 15 groups and the corresponding LOD value ranged from 4 to 31. Ten groups were divided by a lower LOD value of 4 or 5; one group was divided by 9; and another four groups were divided by 25, 30 or 31. One marker was aligned with inappropriate chromosome (marker3869) and 112 markers (8%) revealed significant order errors. Totally there were 1384 markers which were able to be aligned with correct chromosome (Figure 6). The detailed information of 1385 markers was available on Additional file 7.

## Discussion

### 2b-RAD library approach improvement

The 2b-RAD approach was invented by Wang in 2012 and was proven as a simple and flexible method for genome-wide genotyping [14]. However, this technique was rarely used in the published researches. Lacking of pooling library approach as other reduced representation libraries, made it take more manpower and time, and may partly explain the reason.

The existent reduced representation libraries, such as RAD, GBS, two enzyme GBS and double digested RAD, were all pooling library sequencing techniques. The barcode adapters of RAD and double digested RAD were phosphorylated in 5′ end, which made them more expensive in adapter cost than GBS and two enzyme GBS whose adapters were not phosphorylated. Moreover, to mix many libraries for multiplexing sequencing in a flowcell lane, the indexed primer was widely used in Illumina Hiseq platform. For saving library cost, we used the non-phosphorylated adapters and the index primer. The combination of these two methods achieved that using 12 barcode adapters completed the pooling library and multiplexing sequencing rather than using 96 or 48 barcode adapters. However, this approach was not only the method for improving the existent 2b-RAD library approach. The barcode adapters and PCR primers like the style of RAD, GBS, two enzyme GBS or double digested RAD methods could also be modified to fit for 2b-RAD. The method in this research was just a comparatively cost-effective method for materials costs.

For library experiment, the method comprised digestion, ligation, pooling, PCR and size selection. Comparing with Wang’s approach, the method added a size selections procedure, but reduced a step of PCR. The size selection was inevitably, because the fragments between two REs recognition sites could also be ligated with adapter and amplified. They had a large amount reveled by the agarose gel electrophoresis outside 150–200 bp (Additional file 2 - Figure 2).

Anyhow, the improved library approach reduced manpower and time cost. Completing a library including 12 samples took only 5 hours. Achieving 8 libraries containing 96 samples within a day wouldn’t be a problem. So this method was suit for large sample size population.

### The merits and demerits of the method

By filtered step, 33.74% of the raw reads were filtered. The 72.1% of the filtered reads were mapped to reference, indicating that most of these reads may be the fragments between two *Bsa*XI recognition sites. For easy operation, 150–200 bp DNA bands were selected in library procedure. The bands in this region which were less than 160 or more than 164 were probably the fragments between two *Bsa*XI recognition sites and so useless data increased. Using more accurate DNA markers to select the 160–164 bp fragments, could reduce the useless data.

The evenness of each sample reads was important. The reads number differing too big could make analysis difficult and inaccurate. Of the high quality reads, the ratio about the maximum reads numbers divided by minimum reads number was 4.9, showing a high evenness. The sample in each library was only 12, making it easy to keeping the same amount mixing. A library containing 96 samples were not so easy to keep evenness in experimental operation. This was another reason why we used only 12 barcodes for a library. Of course, the barcode adapters could be increased as needed.

The average depth of each *Bsa*XI site was 10.67. For 3598 Stacks markers, 1051 markers (29.2%) were filtered with a higher missing ratio and 1156 markers (32.1%) were filtered by χ^2^ test. For the genetic map result, four groups were divided by a higher LOD value. Some genetics group could not directly gather into a chromosome (chromosome 1, 4 and 9). The genetic group corresponding to chromosome 6 revealed that many markers showed order error. For chromosome 9, fewer markers were assigned to one chromosome arm. Meanwhile, the alignment result of all Stacks markers revealed wide distribution in chromosome (Additional file 6). These demerits indicated that the progeny’s genotypes were not accurate enough. It may attribute to the lower sequencing depth. By the detection of REs sites, we found *Bsa*XI sites were more than *EcoRI*, especially in *Oryza sativa*. The average depth of 10.67 × was a bit lower to confirm heterozygote genotypes. The low-quality data of progeny made that 61.3% of the Stacks marker were filtered and just right most of the markers in one arm of chromosome 9 were filtered. To overcome the shortcomings, the sequencing data should be increased. However, only one marker was aligned to inappropriate chromosome and 112 markers revealed significant order errors, proving the practicability of the approach.

The SNPs between two parents calculated by SOAPsnp were 4769, while by Stacks were 3598. The SOAPsnp was not the professional software for reduced representation genome analysis. It was used for roughly estimating the total SNPs in this research. The comparing results between SOAPsnp and Stacks revealed that the actual SNPs were approximately 3000 to 5000. The markers for genetic map were 1391. Of the published RAD or GBS researches for genetic map analysis, the final marker numbers were from several hundreds to thousands [10, 23–29]. The female parent was Nipponbare, while the male could trace its ancestry to Nipponbare. The two parents had close relationships. However, 3598 markers were obtained which were comparable to published RAD or GBS researches. Using more distantly related parents would receive more SNPs.

The alignment result of Stacks markers revealed that 33 pairs of markers had overlaps. They could be assembled to long tags to discover SNPs. But the probability of the overlapped makers was too low for more valuable applications.

### The practical application in the future

The most important function of reduced representation libraries is genotyping, namely, the marker (main SNPs) discovery. Based on these markers, the genetic map can be constructed, comprising two main applications: QTL fine mapping [23–27] and *de novo* chromosomes assembly [28, 29].

One recognition sites of RAD could produce two tags, making *Hin*dIII had the most simulant tags in 20 species on average. Meanwhile, the RAD tags were long enough to carry out single-end 90 bp sequencing. In addition, RAD had another application - paired end sequencing for *de novo* assembly and marker design [30, 31]. Possessing more and long tags means more chance to obtain SNPs, so RAD was more effective than 2b-RAD in SNP discovery. However, the two tags produced by one RAD REs site located together. Two tags which stand together amount to one marker in linkage map. So marker density for genetic map is mainly depending on the numbers of scattered recognition sites rather than tags. The REs for RAD all possess palindromic recognition sites, as well as *Alf*I, while *Bsa*XI doesn’t. Two kinds of recognition sites result that *Bsa*XI have the most recognition sites, making 2b-RAD approach is potentially more effectively than RAD for constructing high density genetic map. It should be noted that the progeny’s genotypes were not accurate in this research and may be attributed to the lower sequencing depth. The combinations of 4 repeated female samples’ results revealed that doubled data size increased the *Bsa*XI sites coverage to almost 100%. To solve the problem for gaining more accurate data, the one way is to increase sequencing data by doubling lanes. The samples can be reduced to 24 of 2 libraries in a single lane and the total lanes rise to 12. In addition, the design of adapters and PCR primers in this research made 2b-RAD tags could be either performed single-end sequencing or paired-end sequencing (Figure 1). The PE1 and PE2 reads from paired-end 50 bp (PE50) sequencing would be entirely over merged. Hence, except adding flowcell lanes, the other way to double data size is to use paired-end sequencing. In commonly, with a same total data size, a PE lane is more inexpensive than two SE lanes. The paired-end sequencing strategy for 2b-RAD with a significant cost advantage is worth popularizing in the future applications.

The marker density of genetic map is important for *de novo* chromosomes assembly because possessing more markers for a contig or scaffold means more chance to be perfectly assembled. By the assembly simulation, the tiny scaffolds which were potentially perfectly assembled were 28% for *Eco*RI and 47% for *Bsa*XI, indicating 2b-RAD using *Bsa*XI were more effective in chromosomes assembly. Of course, simultaneous use of *Eco*RI and *Bsa*XI markers, the perfect assembly rate rose to 71.3%.

Using several hundreds of DNA markers is common and sufficient for typical QTL mapping studies. Therefore, RAD-QTL approach often use a rare-cutter enzyme (*Sbf*I for instance) to increase read count per RAD tag per individual and reduce sequencing cost per individual [23–25]. Meanwhile, the QTL study using ddRAD - sequencing permitted sequencing of over 1000 individuals in a single HiSeq 2000 lane [13]. The lower requirement of makers in QTL-research for 2b-RAD could be adjusted by two ways. One is to use *Alf*I. The density of *Alf*I sites was nearly 1/3^th^ in plants or 1/2^th^ in animals of *Bsa*XI by simulation. The other way is to use the adapters with 5′-NNF-3′ overhangs (F means a fixed base of anyone of A, T, C or G) that targeted 1/16^th^ (4^4^/4^6^) of all *Bsa*XI sites [14]. The digested fragment of *Alf*I has 2 bp random overhangs on the 3′ ends. For modified adapters with 5′-NF-3′ overhangs for *Alf*I, the targeted sites were also reduced to 1/16^th^ (4^2^/4^4^). An addition comparison of the enzyme sites number was list on Table 3. The result indicated that 1/16^th^ of *Alf*I sites was nearly the same sites of *Sbf*I in plants; while 1/16^th^ of *Bsa*XI sites was nearly the same sites of *Sbf*I in animals. The adapters with the overhangs of “NNT” (T means any two kinds of bases of A, T, C or G) or “NNR” (R means any three kinds of bases of A, T, C or G) also could be used to increase markers than “NNF” if the marker density used by “NNF” adapters are fewer. So the adapter could be flexibly deployed of the fixed bases in its 3′ overhangs according to marker density requirement, making 2b-RAD technique have multiple uses.

**Table 3:** **The enzyme sites comparison between** ***Sbf*** **I,** ***Bsa*** **XI and** ***Alf*** **I**

Species	Size (G)	***Sbf***I	1/16 ^th^ of ***Bsa***XI sites	1/16 ^th^ of ***Alf***I sites
*Arabidopsis thaliana*	0.12	627	2489	788
*Cucumis sativus*	0.24	1197	4078	1485
*Theobroma cacao*	0.33	1572	5113	2488
*Oryza sativa*	0.37	5541	11303	5217
*Vitis vinifera*	0.49	2594	9743	3879
*Cajanus cajan*	0.61	1652	10145	3882
*Solanum tuberosum*	0.73	2957	13256	4378
*Sorghum bicolor*	0.74	11693	23749	9453
*Glycine max*	0.97	3844	18121	8763
*Zea mays*	2.06	50773	71938	27792
Average	0.67	8245	16994	6812
*Drosophila melanogaster*	0.17	2948	3637	3150
*Apis mellifera*	0.23	551	3628	1327
*Anopheles gambiae*	0.27	3164	6286	5009
*Takifugu rubripes*	0.39	20718	13155	6496
*Bombyx mori*	0.48	2041	6050	2958
*Gallus gallus*	1.05	48010	28368	25022
*Danio rerio*	1.41	30677	26637	21215
*Mus musculus*	2.72	60302	83191	37944
*Rattus norvegicus*	2.72	58283	78576	37232
*Homo sapiens*	3.10	77095	80774	36471
Average	1.17	30378	33030	17683

A detailed scheme of how to apply 2b-RAD approach in practice was showed on Table 4. Taking *Oryza sativa* for instance, sequencing of over 1000 individuals in a single HiSeq 2000 lane also can be achieved. Of course, genotyping of this approach can be used for natural population evolutional analysis.

**Table 4:** **The scheme of how to apply I2b-RAD approach**

	High density genetic map	Common – lower density genetic map	
Application	Chromosome assembly	QTL mapping	
Enzyme	*Bsa*XI	*Bsa*XI	*Alf*I
Adapter	Adapter1, adapter2	Adapter1, adapter2 with 5′-NNF-3′ overhangs	Adapter1, adapter2 with 5′-NF-3′ overhangs
Enzyme site percentage	100%	1/16^th^	1/16^th^
The count of samples in a single lane (*Oryza sativa* for instance)	SE50 of 24 samples; PE50 of 48 samples;	SE50 of 384 samples; PE50 of 768 samples;	SE50 of 768 samples; PE50 of 1536 samples;

The count of samples by 1/16^th^ of *Alf*I adapter were calculated based on the simulate data on Table 3. The *Alf*I sites were nearly 1/2^th^ of the *Bsa*XI in *Oryza sativa*.

## Conclusions

By using barcodes adapters, 12 samples were pooled together as one sample to carry on following library procedure. It was not complicated for experiment operators to completing a library including 12 samples within 5 hours. Achieving 8 libraries containing 96 samples within one day wouldn’t be a problem. The improvement of using barcodes adapters made 2b-RAD library preparation become sample and fast. Furthermore, the bioinformatics simulation and F2 population genotyping revealed that 2b-RAD data using *Bsa*XI were effective for high density genetic map. More applications could be achieved by adjustment of enzyme and adapters. So an improved 2b-RAD genotyping approach was established in this research and named as I2b-RAD.

## Electronic supplementary material

Additional file 1: **The adapters and PCR primers sequences.** (XLSX 14 KB)

Additional file 2: **2b-RAD protocol.** (DOCX 91 KB)

Additional file 3: **The windows assembly simulation of** ***Bsa*** **XI and** ***Eco*** **RI.** (XLSX 13 KB)

Additional file 4: **The statistics of experiment reads.** (XLSX 32 KB)

Additional file 5: **The statistics of reads mapping.** (XLSX 41 KB)

Additional file 6: **The markers information derived from Stacks and SOAPsnp.** (XLSX 4 MB)

Additional file 7: **The genetic maps information.** (XLSX 2 MB)
