# Supplementary material for: An improved 2b-RAD approach (I2b-RAD) offering genotyping tested by a rice (Oryza sativa L.) F2 population
Source: BMC Genomics. 2014 Nov 5;15(1):956. doi: 10.1186/1471-2164-15-956 (PMC4236440; doi:10.1186/1471-2164-15-956)
Supplement: Supplementary file 2 — Additional file 2: 2b-RAD protocol. (DOCX 91 KB) [file 12864_2013_6659_MOESM2_ESM.docx]

**2b-RAD protocol**

**Anneal adapters**

Each adapter of dry oligo was eluting in nuclease-free water as a concentration of 100μM. To create double-strand adapter, combine each oligo (top) with its complementary oligo (bottom) in a 1:1 ratio in annealing buffer. The preparation of annealing buffer was listed in table 1. The double-strand adapter was annealed to 25μM using 12.5μL of each pair of oligos and 25μL annealing buffer. In a thermocyler, incubate at 95.0℃ for 10 minutes, and then cool at a rate of not greater than 3°C per minute until the solution reaches a temperature of 25℃. Hold at 4℃. Then the annealed adapters were diluted to a concentration of 5μM.

Table 1: the preparation of annealing buffer

| reagent | volume (μL) |
| --- | --- |
| Tris-HCl (1M, pH 8.0) | 100 |
| EDTA (0.5M, pH 8.0) | 20 |
| NaCl (5M) | 100 |
| nuclease-free water | 9780 |
| total | 10000 |

**Digestion**

The extracted DNA of each sample was adjusted to the concentration of 50ng/μL. The 200ng of high quality genomic DNA was digested. The reaction system was listed in table 2. The reaction was incubated at 37 C for 2h using BsaXI. An additional sample can be digested simultaneously to detect the digestion efficiency by 1% agarose gel electrophoresis. The primary DNA band disappeared and became disperse, indicating a successful digestion (Figure 1).

Table 2: the digestion reaction system

| reagent | volume (μL) |
| --- | --- |
| DNA (50ng/μL) | 4 |
| 10 x buffer 4 | 1 |
| *Bsa*XI (2,000U/mL) | 0.5 |
| nuclease-free water | 4.5 |
| Total | 10 |

Figure 1: The digestion was detected by 1% agarose gel electrophoresis


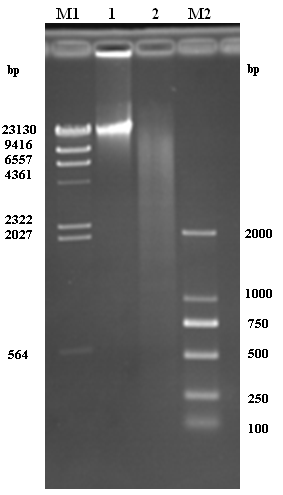


M1: λ-Hind Ш digest (Takara); 1: extracted DNA by CTAB; 2: digested DNA; M2: D2000 DNA Marker (Tiangen)

**Adapter ligation**

The ligation reaction system was listed in table 3. The reaction was incubated at 4℃ for 1 hour, then hold on ice

Table 3: the ligation reaction system

| Reagent | Volume (μL) |
| --- | --- |
| digested mixture | 10 |
| T4 ligase buffer (with ATP, 10 x ) | 2 |
| T4 ligase (400,000 U/mL) | 1 |
| adapter 1 | 1 |
| adapter 2 | 1 |
| nuclease-free water | 3 |
| total | 20 |

**Pooling when purifying**

Twelve samples were completely pooled together to reach an amount of 1~1.5μg. The pooling procedure was carried on when purifying using the QIAquick PCR Purification Kit. The samples with different barcode adapters were gathered to a tube which had been added the wash buffer of purification kit beforehand. Then the pooled DNA was purified according to the manufacturer’s instructions and regarded as a library, eluted in 25μL EB.

**PCR amplication**

The PCR was performed as table 4. Temperature cycling consisted of 98℃ for 30 s followed by 12 cycles of 98℃ for 30 s, 65℃ for 30 s, 72℃ for 30 s with a final *Taq* extension step at 72℃ for 5 min.

Table 4: the PCR system

| Reagent | Volume (μL) |
| --- | --- |
| DNA | 50ng |
| multiplexing PCR primer 1.0 (10μM) | 1 |
| index primer (10μM) | 1 |
| Phusion PCR master mix | 25 |
| nuclease-free water | up to 50 |
| total | 50 |

**Size selection**

The PCR production was detected by 2% agarose gel electrophoresis, then the 150-200bp bands were cut and purified by QIAquick Gel Extraction Kit and eluting in 30μL EB (finger 2).

Figure 2: size selection of 2b-RAD library


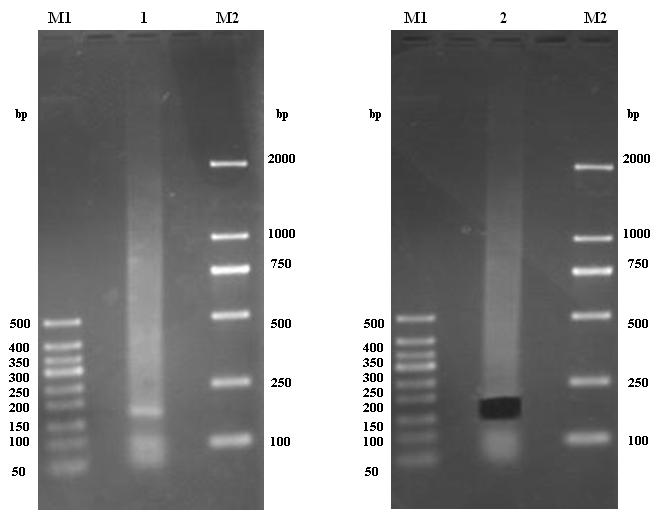


M1: 50bp DNA Ladder (Tiangen); 1: PCR production detected by 2% agarose gel electrophoresis; 2: cut bands; M2: D2000 DNA Marker (Tiangen)
